# The regulation of adipocyte growth in white adipose tissue

**DOI:** 10.3389/fcell.2022.1003219

**Published:** 2022-11-22

**Authors:** Qian Li, Kirsty L. Spalding

**Affiliations:** ^1^ Department of Anatomy, Histology and Embryology, School of Basic Medical Sciences, Department of General Surgery, Huashan Hospital, Fudan University, Shanghai, China; ^2^ Department of Cell and Molecular Biology, Karolinska Institutet, Stockholm, Sweden

**Keywords:** adipocyte, adipocyte hypertrophy, adipose tissue expansion, caveolae, obesity, endoreplication, lipogenesis, hypoxia

## Abstract

Adipocytes can increase in volume up to a thousand-fold, storing excess calories as triacylglycerol in large lipid droplets. The dramatic morphological changes required of adipocytes demands extensive cytoskeletal remodeling, including lipid droplet and plasma membrane expansion. Cell growth-related signalling pathways are activated, stimulating the production of sufficient amino acids, functional lipids and nucleotides to meet the increasing cellular needs of lipid storage, metabolic activity and adipokine secretion. Continued expansion gives rise to enlarged (hypertrophic) adipocytes. This can result in a failure to maintain growth-related homeostasis and an inability to cope with excess nutrition or respond to stimuli efficiently, ultimately leading to metabolic dysfunction. We summarize recent studies which investigate the functional and cellular structure remodeling of hypertrophic adipocytes. How adipocytes adapt to an enlarged cell size and how this relates to cellular dysfunction are discussed. Understanding the healthy and pathological processes involved in adipocyte hypertrophy may shed light on new strategies for promoting healthy adipose tissue expansion.

## Introduction

Adipose tissue plays a vital role in the regulation of whole-body energy homeostasis, sequestering, storing and releasing lipids as needed. Adipose tissue lipid storage capacity can be increased by enlarging fat cell volume or increasing fat cell number. Increased fat cell number (hyperplastic expansion) generates new small-sized adipocytes and is considered to offer a healthy mechanism of adipose tissue expansion (Vishvanath and Gupta, 2019). Considerable enlargement of cell size (hypertrophic expansion), however, leads to adipocyte dysfunction and has been widely recognized as the primary factor in the development of obesity-related metabolic syndrome (Weyer et al., 2000; Skurk et al., 2007; Laforest et al., 2015; Suarez-Cuenca et al., 2021; Honecker et al., 2022).

Mature adipocyte size varies greatly with a broad range in cell diameter, ranging from 20 µm to several hundred µm in humans (Fang et al., 2015; Honecker et al., 2021). No size range for adipocytes is universally accepted to define large versus small fat cells, with size cut-offs and cell measurement methods varying between studies (Ye et al., 2022). A meta-analysis, looking at more than 89 studies where adipocyte size data from humans was included, reported a mean adipocyte diameter of 75–80 µm for lean individuals (based on collagenase-digested quantification of subcutaneous adipocytes), with adipocyte size increasing with BMI and plateauing at approximately 120 µm in severely obese individuals (Ye et al., 2022). This is in line with Stenkula and Erlandson-Albertsson who propose an adipocyte diameter of less than 70 µm be considered small, 70–120 µm large and greater than 120 µm very large (Stenkula and Erlanson-Albertsson, 2018).

Studies comparing small versus large adipocytes from the same individual have contributed significantly to a better understanding of the functional consequences of adipocyte hypertrophy. Large adipocytes display distinct differences in gene expression related to inflammation, mitochondrial dysfunction and fatty acid metabolism (Jernas et al., 2006; Honecker et al., 2022). Hauner and colleagues fractionated adipocytes into small, medium, large and extra-large size bins, demonstrating that large adipocytes secrete increased levels of interleukin-6 (IL-6), interleukin-8 (IL-8), monocyte chemoattractant protein-1 (MCP-1) and leptin, further linking hypertrophic adipocytes to adipose tissue inflammation (Skurk et al., 2007). Functionally, an attenuated insulin response, including defects in glucose transporter type 4 (GLUT4) trafficking to the cell membrane, has been shown for hypertrophic adipocytes *in vitro* (after inducing adipocyte hypertrophy by feeding with long-chain fatty acids) as well as *in vivo* (protein analysis evaluated by immunofluorescence microscopy when comparing large versus small human adipocytes) (Franck et al., 2007; Kim et al., 2015).

In addition to studies investigating large versus small adipocytes from the same individual, average adipocyte size differences between individuals has also yielded important insights into the cellular dysfunction of large adipocytes and their contribution to metabolic health. In monozygotic twins discordant for BMI, Pietiläinen and colleagues report that adipose tissue expansion in the ‘obese’ twin occurs predominantly *via* hypertrophic adipose tissue expansion, with an accompanying decrease in the expression of mitochondrial genes and an increase in the expression of genes regulating cell death and inflammation (Heinonen et al., 2014; Heinonen et al., 2017). Hypertrophic expansion is also characterized by reduced responsiveness to insulin, resulting in decreased glucose uptake, increased basal lipolysis and blunted stimulated lipolysis (Salans et al., 1974; Laurencikiene et al., 2011). Furthermore, adipocyte enlargement limits oxygen diffusion, initiating regional hypoxia and adipose tissue fibrosis (Hosogai et al., 2007; Lawler et al., 2016). This in turn constrains adipose tissue expansion and adipocyte lipid storage capacity (Marcelin et al., 2019). Fatty acids that are not efficiently stored in adipocytes end up being deposited into ectopic tissues (such as muscle, liver, heart and pancreas), causing lipotoxicity and impaired systemic insulin sensitivity (Virtue and Vidal-Puig, 2010; Taylor et al., 2018; Singh et al., 2019; Lytrivi et al., 2020).

Adipocyte hypertrophy is recognized as a risk factor predicting reduced insulin sensitivity (Arner et al., 2010; Fang et al., 2015; McLaughlin et al., 2016). Whilst a large number of studies have focused on the health consequences of adipocyte hypertrophy, less attention has been paid to the remarkable ability of adipocytes to undergo extreme changes in size during their lifetime, without the development of cellular dysfunction. Adipocyte growth *per se* is a healthy and necessary mechanism whereby adipocytes accommodate nutrient overload. Dramatic cytoskeletal rearrangement occurs during adipocyte enlargement, with cytoskeleton components like filamentous-actin, septin, and vimentin significantly upregulated in obesity (Moreno-Castellanos et al., 2017; Hansson et al., 2019; Kim et al., 2019; Roh et al., 2020). Increased transcript and protein content are also required to support enhanced lipogenic and adipokine secretory activity in enlarged adipocytes (Jamdar and Osborne, 1981; Farnier et al., 2003). Despite this amazing capacity to accommodate significant changes in cell size, extended adipocyte hypertrophy results in an inability to handle nutrient overload and maintain growth-related homeostasis, contributing further to adipocyte hypertrophy and related adipose tissue dysfunction. In the following sections we discuss several mechanisms whereby the adipose tissue regulates healthy fat cell expansion, including lipogenesis, lipolysis, cell membrane dynamics, growth activating pathways and vascular dynamics. Understanding the mechanisms underlying the healthy expansion of fat cells, may contribute to a clearer understanding of the molecular mechanisms contributing to hypertrophic adipocyte dysfunction and suggest novel interventions for obesity-related disorders.

### Lipogenesis regulated adipocyte expansion

**FIGURE 1:**
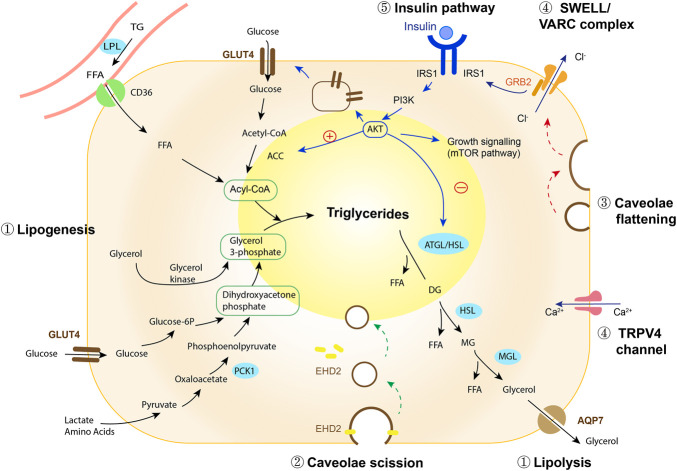
Regulation of adipocyte expansion. ① Lipid droplet growth is mediated *via* the balance between lipogenesis and lipolysis (black arrows, and see Supplementary Box S1). Insulin plays a role in promoting lipogenesis while inhibiting lipolysis, keeping lipid stored in adipocytes. ② EHD2 stabilizes caveolae on the cell membrane and disassembly of EHD2 facilitates caveolae to detach from the cell membrane, transporting the lipid-enriched membrane into the cytosol where it integrates into the lipid droplet (green arrows). ③ In response to mechanical stretch or osmotic pressure (red arrows), caveolae flatten, increasing cell membrane area. ④ Lipid droplet expansion and caveolae flattening activate SWELL/VARC ion channel complexes causing calcium ion efflux. TRPV4 ion channels sense changes in intracellular volume induced by lipid droplet enlargement, activating calcium ion influx. The activation of ion channels adjusts osmotic pressure and facilitates the cell to swell. In addition, the flattening of caveolae triggers a restructuring of the SWELL1/VRAC complex, destabilizing GRB2-IRS1 interactions and activating insulin-mediated growth signaling. ⑤ Insulin regulates lipogenesis and lipolysis (blue arrows), whilst also mediating activation of growth and mitogenic pathways, in order to accommodate the increased protein and transcript demands required for cell growth. Abbreviations: ACC, acetyl-CoA carboxylase; AKT, protein kinase B; AQP7, aquaglyceroporin-7; ATGL/HSL, adipose triglyceride lipase/hormone-sensitive lipase; EHD2, EH-domain–containing protein 2; FFA, free fatty acid; glucose-6-p, glucose-6-phosphatase; GLUT4, glucose transporter type 4; GRB2, growth factor receptor bound 2; IRS1, insulin receptor substrate 1; LPL, lipoprotein lipase; MGL, monoacylglycerol lipase; PCK1, phosphoenolpyruvate carboxykinase; PI3K, phosphatidylinositol-3-kinase; TG, triglyceride; TRPV4, transient receptor potential vanilloid 4.

Lipid droplet enlargement in obesity is the result of increased lipid storage and/or reduced lipid breakdown (lipolysis), resulting in increases, or decreases, in fat cell size. During the process of lipogenesis, adipocytes utilise fatty acids and glucose to synthesise triglycerides, which are sequestered into the lipid droplet (Figure 1, Supplementary Box S1). The major resources for lipogenesis are circulating triglycerides which are transported *via* liver-derived very low-density lipoprotein (VLDL) complexes or from intestinal-derived chylomicrons. Since adipocytes cannot directly take up triglycerides, lipoprotein lipase (LPL) bound to the capillary endothelium assists in the hydrolysis of triglycerides into fatty acids, which are then transported into adipocytes. In this process, LPL is the crucial rate-limiting enzyme determining circulating lipid availability for lipogenesis (Weinstock et al., 1997).

Besides the availability of fatty acids, the rate of glycerol-3-phosphate (G3P) synthesis limits fatty acid esterification, and thus affects triglyceride formation. Glyceroneogenesis, the process whereby cells utilize pyruvate, lactate and alanine to generate G3P, is considered as the major resource for adipocyte G3P, in both fasting and feeding states (Nye et al., 2008). Phosphoenolpyruvate carboxykinase 1 (PCK1) catalyzes oxaloacetate to phosphoenolpyruvate and is the crucial regulatory step in glyceroneogenesis. Enhancement of glyceroneogenesis, through overexpression of PCK1 in mice, increases fatty acid esterification and promotes triglyceride synthesis. This induces adipocyte enlargement, whilst maintaining low circulating fatty acid levels and preserving whole-body insulin sensitivity (Franckhauser et al., 2002). Conversely, the downregulation of glyceroneogenesis, through the inactivation of PCK1, limits fatty acid re-esterification and fat accumulation, resulting in lipodystrophy and profound systemic insulin resistance (Chaves et al., 2006; Millward et al., 2010). As such, glyceroneogenesis is considered one of the key players in the maintenance of triacylglycerol stores and adipocyte size.

Glycerol derived from lipolysis can be recycled and phosphorylated *via* glycerol kinase to serve as another resource for G3P synthesis. The membrane glycerol channel aquaporin 7 (AQP7) facilitates glycerol efflux and regulates intracellular glycerol levels. AQP7 knockout in mice leads to adipocyte glycerol accumulation, elevated glycerol kinase activity and increased triacylglycerol accumulation, suggesting an important role for glycerol recycling in adipocyte expansion (Hibuse et al., 2005). Upregulated glycerol kinase activity has been observed in murine adipose tissue after high-fat diet feeding (HFF) as well as in individuals with obesity, when compared to lean individuals (Chakrabarty et al., 1984; Iena et al., 2020). Of note, human data is less consistent than animal studies, with confusion surrounding the expression and localization of aquaglyceroporins in adipose tissue (Mourelatou et al., 2019; Huang et al., 2022; Iena et al., 2022) and sex-dependent differences impacting experimental conclusions (Lebeck et al., 2012; Lebeck et al., 2018). In summary, AQP7 facilitates the release of adipose tissue glycerol, which in turn regulates adipose tissue triglyceride synthesis *via* controlling glycerol availability and glycerol kinase activity (Iena et al., 2022). Decreased AQP7 associates with reduced lipolysis, which together with activated glycerol kinase enhances glycerol recycling, promoting adipocyte hypertrophy.

Adipose tissue lipid storage capacity is enhanced in obesity. Proteins that are responsible for lipid uptake, including the scavenger receptor protein CD36 (Bonen et al., 2006; Grzegorczyk et al., 2018) and fatty acid transport protein (FATP) family members (Gertow et al., 2004) are significantly upregulated in obesity, facilitating lipid influx. Total glycerolipid biosynthesis rate, evaluated by ^14^C acetate incorporation into different lipid fractions, was higher in obese rats compare to lean and attributed to enhanced lipid synthesis (Jamdar and Osborne, 1981). In line with this, the rate of triglyceride storage (the net amount of lipid stored in the adipose tissue per year) is markedly increased in individuals with obesity compared to lean individuals (Arner et al., 2011). As obesity progresses, the development of hyperinsulinemia can increase adipose tissue lipogenesis, further promoting lipid storage (Assimacopoulos-Jeannet et al., 1995; Koopmans et al., 1999). Together this data supports a role for persistent, augmented lipogenesis acting as an intrinsic force driving adipocyte growth.

In contrast, *de novo* lipogenesis (DNL) is dependent on adipocyte glucose transport and is suppressed in hypertrophic adipocytes and obesity (Roberts et al., 2009; Eissing et al., 2013). Impairments in GLUT4 expression and glucose uptake markedly suppress DNL, as observed in obese insulin resistant individuals (Ortega et al., 2010; Eissing et al., 2013). As such, DNL is an unlikely contributor to lipid droplet expansion. Accumulating evidence, however, suggests that the bioactive lipid products of DNL are essential for optimal adipocyte cellular membrane fluidity, increased insulin sensitivity and improved systemic metabolic homeostasis (Collins et al., 2010; Herman et al., 2012; Vijayakumar et al., 2017). The blunted DNL capacity of hypertrophic adipocytes also impacts on the production of metabolically beneficial lipids, such as insulin-sensitizing palmitic acid-hydroxy stearic acids (PAHSAs), resulting in decreased systemic insulin sensitivity (Yore et al., 2014; Hammarstedt et al., 2018; Hsiao and Guertin, 2019).

### Lipolysis regulated adipocyte expansion

The average age of triglycerides in subcutaneous human adipocytes is 1.6 years, demonstrating that adipocyte lipid turns over approximately 6 times during the lifespan of a fat cell (Spalding et al., 2008; Arner et al., 2011). These, and subsequent radiocarbon studies, show that adipocyte lipid removal rate (lipolysis followed by oxidation) differs depending on a number of parameters, such as person age, sex, BMI and fat depot (Spalding et al., 2017; Arner et al., 2019). These retrospective studies across the lifespan demonstrate that triglyceride removal rate in human adipocytes is a dynamic and regulated process (Arner et al., 2011).

Lipolysis is the biochemical pathway in which triglycerides in the lipid droplet are broken down to liberate glycerol and three molecules of fatty acids (Figure 1). Obesity is associated with increased spontaneous (basal), but decreased hormone-stimulated fat cell lipolysis. Studies using microdialysis to measure the release of the lipolytic substrate glycerol, reveal a higher basal lipolytic rate in obese individuals compared to lean (Jansson et al., 1992). No difference is observed when normalized to adipose tissue mass, suggesting that basal lipolysis increases in proportion to fat mass (Jansson et al., 1992; Reynisdottir et al., 1994). In humans, subcutaneous adipocytes are larger and have a higher basal lipolytic rate than visceral adipocytes (Tchernof et al., 2006). This is somewhat paradoxical given that a higher lipolytic rate would be expected to associate with a decreased fat cell size. Development of obesity, however, is characterized by an overall decrease in triglyceride removal rate (Arner et al., 2011) and it is proposed that downregulation in stimulated lipolysis is one of the major factors contributing to body weight gain (Arner et al., 2018). Stimulated lipolysis can be triggered by hormones, such as catecholamines, cortisol and glucagon (Duncan et al., 2007). Catecholamine-stimulated lipolysis occurs through the activation of β-adrenergic receptors (ADRBs) on the plasma membrane, representing the major endogenous stimulation of lipolysis in humans. Individuals with obesity exhibit an almost 70% reduction in ADRB2 sensitivity (Reynisdottir et al., 1994; Schiffelers et al., 2001) and a 50% reduction in the number of ADRB2 positive adipocytes in obesity and hyperinsulinemia (Hagberg et al., 2018). This data thus suggests a heterogenous lipolytic response of adipocytes to catecholamine stimulation in obesity and hyperinsulinemia. ADRB3, the predominant subtype of adrenergic receptor mediating adipocyte lipolysis in mice, has also been found to play an important role in regulating adipocyte lipolysis and thermogenesis in humans (Antraco et al., 2021; Cero et al., 2021). Similar to ADRB2, ADRB3 expression decreases in adipocytes in patients with obesity and represents a mechanism whereby adipocytes become resistant to catecholamines in obesity (Valentine et al., 2022). Supporting this, a longitudinal study by Rydén and colleagues showed that inefficient lipolysis (high basal/low stimulated) contributed to increased lipid accumulation in obesity, predicting future weight gain and development of insulin resistance (Arner et al., 2018).

### Caveolae and membrane ion channel regulated adipocyte growth

Lipid accumulation leads to large-scale changes in adipocyte volume. A short-term overfeeding study by Cushman and colleagues showed that a ∼3 kg increase in body weight in moderately obese individuals, resulted in a 10% increase in adipocyte size (McLaughlin et al., 2016). In order to accommodate this, adipocytes require feedback mechanisms which enable them to detect size changes and activate growth pathways. Several complexes located on the adipocyte cell membrane can sense mechanical or osmotic forces resulting from lipid droplet enlargement (Thorn et al., 2003; Ye et al., 2012; Zhang et al., 2017). This in turn activates concomitant adaptive responses, with caveolae an essential part of this response.

Caveolae are 25–150 nm diameter plasma membrane microdomains, which invaginate to the cytosol (Thorn et al., 2003). Due to their flask-like design, caveolae serve as a reserve for the membrane, unfolding and incorporating into the cell surface membrane in response to stretch or swell stimulation (Kozera et al., 2009) (Figure 1). Adipocytes have an extremely high abundance of caveolae (Thorn et al., 2003). It is estimated that approximately one million caveolae are localized to the plasma membrane of a 100 μm diameter adipocyte in rats, potently increasing the available surface area of the plasma membrane by 50% (Thorn et al., 2003). In the case of nutrient overload, adipocyte growth and membrane expansion requires continuous caveolae formation (Briand et al., 2014). The density of caveolae on the cell membrane is maintained during adipocyte growth. Two essential caveolar proteins, caveolin-1 and cavin-1, are significantly upregulated in hypertrophic adipocytes, with expression levels correlating linearly with adipocyte surface area (Hulstrom et al., 2013). In contrast, decreasing lipid droplet size by prolonged stimulation of lipolysis, strikingly reduces the number of caveolae invaginated on the plasma membrane (Briand et al., 2014). Cholesterol is an essential component of caveolae. Upon adipocyte expansion cholesterol from the plasma membrane redistributes to the lipid droplet, resulting in a comparative depletion of membrane cholesterol in hypertrophic human adipocytes (Le Lay et al., 2001). Insufficient plasma membrane cholesterol impairs caveolae-dependent insulin signaling and the recruitment of GLUT4 to the membrane, leading to insulin resistance in hypertrophic adipocytes (Parpal et al., 2001; Karlsson et al., 2004).

Besides a role in cellular membrane expansion, it was recently shown that caveolae detach from the plasma membrane (scission) and act like a vesicle, trafficking to intracellular organelles to facilitate lipid uptake and enhance lipid droplet growth (Hao et al., 2020; Matthaeus et al., 2020). CD36 is an important fatty acid transporter which is highly abundant in adipocyte caveolae structures. Caveolae-mediated fatty acid uptake can be initiated by the binding of long-chain fatty acids to CD36, which triggers caveolae scission and endocytosis (Hao et al., 2020). In line with this, Lundmark and colleagues showed that lipid composition in caveolae plays an important role in regulating caveolae scission dynamics: A small increase in glycosphingolipids and cholesterol results in the selective accumulation of these lipids in caveolae structures, leading to a reduction in the amount of EH-domain–containing protein 2 (EHD2), stimulating caveolae to bud from the plasma membrane (Hubert et al., 2020). EHD2, which is located on the neck of caveolae, restrains caveolae to the membrane (Hoernke et al., 2017). Loss of EHD2 increases caveolae scission dynamics and mobility, promoting the lipid-enriched cell membrane to travel into the cytosol through caveolae endocytosis, contributing significantly to lipid droplet enlargement (Hao et al., 2020; Matthaeus et al., 2020). In human adipose tissue, EHD2 expression is reduced in obese individuals (Matthaeus et al., 2020). Whether EHD2-mediated caveolar dynamics are altered in human adipocytes and if this contributes to lipid droplet enlargement, as suggested by *in vitro* studies (Matthaeus et al., 2020) requires further investigation (Fryklund et al., 2021).

Lipid droplet enlargement changes fat cell intracellular volume, altering the mechanical and osmotic properties of adipocytes. These changes are sensed by complexes located on the fat cell membrane, activating a cascade of events designed to help the adipocyte adapt to cell growth. Transient receptor potential vanilloid 4 channel (TRPV4) is a cell volume sensor activated by growth-created swell-stimuli to regulate calcium ion homeostasis in the cytoplasm (Sanchez et al., 2016; Lee et al., 2019; Toft-Bertelsen and MacAulay, 2021). *In vitro* studies on differentiated adipocytes show that inducing adipocytes to swell (by exposing adipocytes to a hypotonic environment), triggers adipocyte cell membrane depolarization and Ca2+ entry through activation of TRPV4 channels (Ye et al., 2012; Sanchez et al., 2016). Increased intracellular Ca2+ induces the rapid phosphorylation of extracellular signal-regulated kinase 1/2 (ERK1/2), activating an important mitogenic/growth pathway.

SWELL1 (also known as LRRC8A, leucine rich repeat containing protein 8a) is a multipass transmembrane protein which forms an essential component of the volume-regulated anion channel (VRAC) (Qiu et al., 2014). Mechanical stimulation, caused by lipid droplet growth, results in caveolae flattening on the adipocyte plasma membrane, triggering a restructuring of the SWELL1/VRAC complex (Gunasekar et al., 2019). In normal conditions, SWELL1, through its C-terminal leucine-rich repeat domain, connects with growth factor receptor bound 2 (GRB2) to bind with insulin receptor substrate 1 (IRS1) and negatively regulate insulin signaling (Zhang et al., 2017; Gunasekar et al., 2019; Gunasekar et al., 2022). Lipid droplet expansion changes the morphology of caveolae and creates mechanical stretch, destabilising GRB2-IRS1 interactions and releasing suppression of insulin-PI3K-AKT2 signaling (Zhang et al., 2017; Gunasekar et al., 2022). The activated insulin growth pathway promotes lipogenesis and lipid droplet expansion, supporting continued adipocyte growth and potentiating adipocyte hypertrophy during nutrient overload. Thus, intrinsic mechanic force resulting from an expanded lipid droplet is sensed by caveolae and through activation of SWELL1/VRAC, triggers a cascade of growth signaling events (Figure 1). Taken together these data demonstrate the important role of caveolae and swell-sensor complexes in the plasma membrane in facilitating adipocyte growth and the healthy expansion of human adipose tissue.

### Growth activating pathways

Adipocyte enlargement results from an increase in lipid droplet size. The enlarged lipid droplet places increasing demands on protein production to facilitate plasma and lipid droplet membrane growth. In addition, hypertrophic adipocytes face increased lipogenic, lipolytic and endocrine demands, placing increased stress on the cell and necessitating the need to activate growth signaling pathways (Farnier et al., 2003; Laurencikiene et al., 2011). The mechanistic target of rapamycin (mTOR), and especially mTOR complex 1 (mTORC1), is a highly conserved pathway regulating a variety of processes contributing to cell growth and is suggested to be involved in adipocyte size regulation (Valentinis et al., 2000; Saxton and Sabatini, 2017). Insulin and insulin like growth factor-1 can activate mTORC1 through the phosphorylation of AKT (also known as protein kinase B, PKB) and inhibit the mTORC1 negative regulator, the tuberous sclerosis protein complex (TSC). As a nutrient-sensing complex, mTORC1 senses cellular amino acid content through heterodimeric Rag GTPases and vacuolar protein sorting 34 -phospholipase D1 pathways (Yoon and Choi, 2016; Carroll et al., 2017). In response, mTORC1 activates the downstream substrates p70S6 Kinase 1 (S6K1) and eIF4E binding protein, promoting protein synthesis, *de novo* lipid synthesis for plasma membrane expansion and nucleotide synthesis required for DNA replication (Saxton and Sabatini, 2017).

As a key regulator in maintaining adipose mass, mTORC1 has been implicated in regulating adipocyte maturation (adipogenesis) (Carnevalli et al., 2010) and lipid storage, including the activation of sterol regulatory element-binding protein (SREBP) to promote lipogenesis and inhibit lipolysis (Ricoult and Manning, 2013; Lee et al., 2016; Crewe et al., 2019). Raptor (regulatory-associated protein of mTOR) is an adaptor protein that associates with mTOR to negatively regulate mTOR kinase activity. In rodents, mTORC1 deactivation *via* adipocyte-specific raptor knockout downregulates the expression of peroxisome proliferator-activated receptor γ (PPARγ), reduces glyceroneogenesis and triglyceride synthesis and increases fatty acid release through insufficient suppression of lipolysis. This leads to lipodystrophy and ectopic lipid deposition in the liver (Lee et al., 2016; Paolella et al., 2020; Andrade et al., 2021), suggested a crucial role of mTORC1 in promoting adipocyte growth and fat accumulation.

It is well established that insulin plays a pivotal role in promoting adipocyte growth in insulin-sensitive adipocytes (Cignarelli et al., 2019). Less understood is how hyperinsulinemia and insulin resistance in adipocytes affect adipocyte growth. It has been suggested that different arms of the insulin signaling pathway are selectively blunted in insulin resistant adipocytes, with glucose uptake primarily affected and other AKT signaling nodes remaining intact (Tan et al., 2015). Chronic hyperinsulinemia may overstimulate and potentially trigger hyperactivation of downstream insulin effectors such as mTORC1. This hypothesis, which has been explored in the liver, vasculature and brain, is referred to as the “paradox of selective insulin resistance” (Brown and Goldstein, 2008; Ferris and Kahn, 2016; Kubota et al., 2017). Preserved insulin signaling, with phosphorylation of the insulin receptor and AKT (T473) was not impaired in adipocytes from individuals with obesity and hyperinsulinemia, despite a significant downregulation of GLUT4 (Li et al., 2021). The expression of S6K, an mTORC1 downstream effector, is upregulated at a transcript level in visceral adipose tissue in individuals with obesity and insulin resistance (Catalan et al., 2015). Moreover, the expression of a key cell cycle regulator and downstream target of mTORC1, cyclin D1, is upregulated in obese individuals and significantly correlates with adipocyte size (Li et al., 2021). Together, the data supports activation of the AKT-mTORC1 pathway in response to insulin, even in whole body and adipocyte insulin resistance.

### Cell cycle regulated adipocyte growth

High levels of insulin, growth factors or hormones can act as strong growth stimulators, inducing cell enlargement and proliferation (Vander Heiden et al., 2001; Hodge et al., 2004; Byron et al., 2006; Beith et al., 2008; Celton-Morizur et al., 2009). Prolonged mitogenic signalling, such as chronic insulin exposure (hyperinsulinemia), has recently been shown to induce cell cycle re-entry in postmitotic cells, including hepatocytes (Celton-Morizur et al., 2009; Wang et al., 2017), beta cells (Aguayo-Mazzucato et al., 2019), neurons (Frade and Ovejero-Benito, 2015) and adipocytes (Li et al., 2021), with cell cycle re-entry in post-mitotic cells associated with cellular growth (Tamori and Deng, 2013; Edgar et al., 2014).

**FIGURE 2:**
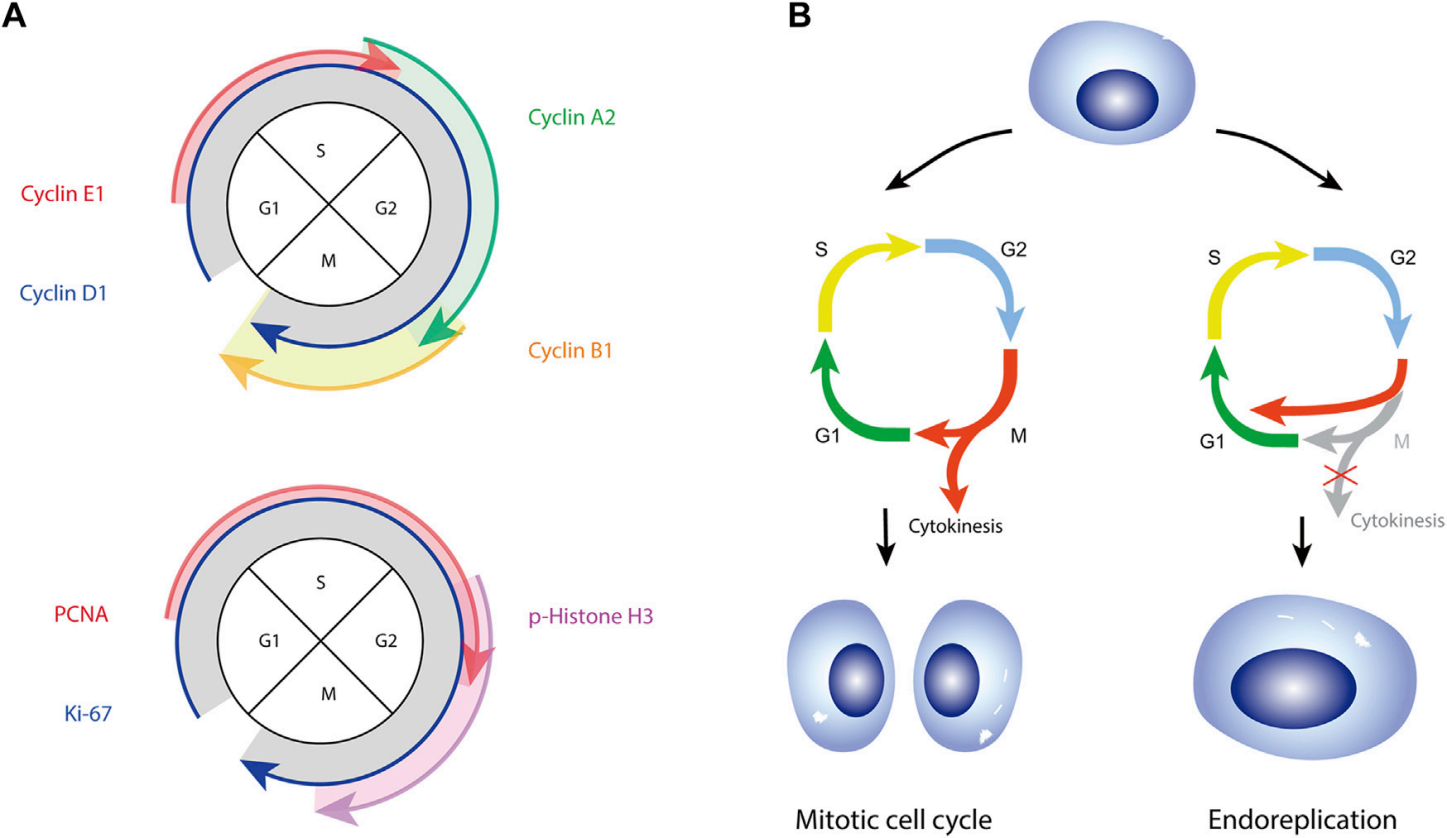
**(A)** Schematic of cyclin proteins (top panel) and proliferation markers (bottom panel) expressed in the four phases of a mitotic cell cycle. **(B)** Schematic representation of mitotic and endoreplicative cell cycles. Both cell cycle programs include phases of growth (G1 and G2) and DNA synthesis (S), however only a mitotic cell passes through M-phase and undergoes cytokinesis. A mitotic cell cycle gives rise to two diploid daughter cells. Endoreplicating cells replicate DNA but skip M-phase to return to G1, resulting in mononucleated polyploid cells (endocycle)‐as demonstrated for adipocytes (Li et al., 2021) and shown in the figure, or pass through M-phase but fail to undergo cytokinesis, resulting in diploid binucleated cells (not shown).

How adipocytes respond to strong growth stimuli remains poorly examined and not fully understood. Several studies have observed the expression of cell proliferation markers, Ki-67, proliferation cell nuclear antigen (PCNA), phospho-histone H3 (pHH3) and anillin in white and brown human adipocytes (Rigamonti et al., 2011; Fukano et al., 2016; Li et al., 2021; Bailey et al., 2022). Cell cycle related proteins have also been reported in mature adipocytes (Kim et al., 2001; Li et al., 2021; Park et al., 2021). This includes the expression of cyclins which are well known to drive cell cycle progression, through the formation of complexes with their corresponding cyclin dependent kinases (CDKs) regulating a myriad of substrates, e.g., cyclins D1, E1, and A2 (Figure 2A). Fajas and colleagues revealed both mouse and human mature adipocytes express cyclin D3 and CDK4, with transcript levels of cyclin D3 positively correlating with BMI (Lagarrigue et al., 2016). Protein levels of cyclin D1 have also been shown to be upregulated in mature adipocytes differentiated *in vitro* (Kim et al., 2001). Cyclin D1 can impact lipid metabolism, with cyclin D1 downregulation significantly inhibiting the expression of lipid synthesis-related genes (C/ebpα, Scd1 and Dgat2) in zebrafish liver cells (Ding et al., 2021). CDK4 in adipocytes was found to promote insulin sensitivity by phosphorylating IRS-2, inducing lipogenesis and inhibiting lipolysis, leading to increased fat mass and adipocyte hypertrophy (Lagarrigue et al., 2016). Adipocyte nuclear size has also been shown to correlate positively with adipocyte cell size, indicating a link between adipocyte cell cycle re-entry and adipocyte hypertrophy (Li et al., 2021). In summary, these studies demonstrate an ability of postmitotic mature adipocytes to activate a cell cycle program in response to a mitogenic signal, facilitating mature adipocyte growth.

Cell cycle entry observed in mature mouse adipocytes or 3T3L1 cells prompted the hypothesis that adipocytes can proliferate (Rigamonti et al., 2011; Fukano et al., 2016; Bailey et al., 2022). In human mature adipocytes Spalding and colleagues found no evidence for adipocyte proliferation and instead argue that adipocytes activate an endoreplicative rather than mitotic cell cycle (Li et al., 2021) (Figure 2B). Endoreplication, also referred as a “postmitotic” cell cycle (Levine, 2004), is a commonly adopted mechanism whereby cells increase nuclear DNA content as a mechanism to accommodate large increases in cell size (Edgar et al., 2014; Gentric and Desdouets, 2014; Gandarillas et al., 2018). During endoreplication, fully differentiated cells re-enter cell cycle and synthesise DNA, but do not undergo cytokinesis and divide (Figure 2B). This results in large cells with increased nuclear size and DNA content (endoreplication), and/or nuclear number (endomitosis). We recently demonstrated that mature human adipocytes, despite long being considered postmitotic, respond to obesity and hyperinsulinemia by activating a cell cycle program, with a concomitant increase in cell and nuclear size and nuclear genetic content (Li et al., 2021). Endoreplication, unlike mitotic cell division, does not involve cytoskeletal rearrangement, ultimately causing less disruption to highly structured tissues (Edgar and Orr-Weaver, 2001). Increased DNA content in endoreplicated cells is believed to enhance cellular capacity to produce transcripts and proteins to permit cell growth and facilitate nutrient transport and storage (Gandarillas et al., 2018). Endoreplication is thus considered an advantageous strategy for cells needing to adapt to continuous growth. The recent description of endoreplication in human adipocytes necessitates further studies to better understand the exact role of polyploidy in promoting and regulating adipocyte cell growth in healthy and pathological settings.

### Adipose tissue vasculature

Adipocyte growth is linked to increased oxygen consumption, which induces adipose tissue hypoxia and stimulates vasculature remodeling in an attempt to maintain oxygen levels (Lee et al., 2014; Caputo et al., 2021). It has been shown that 3 days of HFF already elevates circulating fatty acid levels. Elevated fatty acid induced adenine nucleotide translocase 2 (ANT2) mediates proton leakage from the mitochondrial intermembrane space into the mitochondrial matrix, resulting in an increase in uncoupled respiration and oxygen consumption, leading to a comparatively intracellular hypoxic state (Lee et al., 2014). Intracellular hypoxia induced at the onset of obesity efficiently upregulates the hypoxic master transcriptional regulator, HIF-1α (hypoxia-inducible factor–1α), as well as the potent angiogenic factor VEGF (vascular endothelial growth factor) (Lee et al., 2014; Seo et al., 2019). Gilardi and colleagues compared early and late adipose tissue responses to HFF in mice and revealed an association between adipocyte size and angiogenic response (Caputo et al., 2021): Visceral white adipose tissue (WAT) contained large adipocytes and angiogenic signaling was initiated following 1 week of HFF; subcutaneous adipocytes, however, first underwent cell size expansion and subsequently initiated angiogenesis following 8 weeks of HFF (Caputo et al., 2021). The data suggests that upon a threshold size of healthy adipocyte growth, further fat cell growth is mediated through activation of the vasculature, followed by the commitment of adipocyte progenitors and adipogenesis (hyperplastic expansion) (Caputo et al., 2021). Indeed, remodelled vasculature not only supplements oxygen levels, but also increases the population of white adipocyte progenitors which reside in the mural compartment of vascular structures, directly contributing to the maintenance and expansion of adipose tissue (Tang et al., 2008; Gupta et al., 2012; Vishvanath et al., 2016; Jiang et al., 2017).

As nutrient overload continues, adipocyte size is further increased, however angiogenesis becomes impaired due to an imbalance in the secretion of angiogenic regulators (Corvera and Gealekman, 2014). Transcript expression of the most potent pro-angiogenic factor, VEGF, is reduced by almost 60% and capillary density reduced by 44% in overweight/obese individuals, compared with lean individuals (Pasarica et al., 2009). Transforming growth factor-β1 (TGF-β1) is an anti-angiogenic factor and is significantly increased in obese adipose tissue in both mice and humans (Samad et al., 1997; Fain et al., 2005). In addition to its role in limiting vascular formation, TGF-β1 can promote feeding-associated adipocyte lipogenesis, facilitating fat accumulation and adipocyte growth (Lee et al., 2021; Toyoda et al., 2022). Dysregulated pro- and anti-angiogenic molecules impair angiogenesis, resulting in a significant decrease in vascular density, as observed in the adipose tissue of obese adipose individuals (Bolinder et al., 2000; Caputo et al., 2021). Low vasculature density exacerbates adipocyte hypoxia, which is strongly linked to adipose tissue inflammation and dysfunction. Different from that seen in short-term HFF, chronic hypoxia-induced HIF-1a upregulation fails to induce VEGF expression in severely obese animals. Instead, HIF-1α stimulates the secretion of inflammatory chemokines, such as MCP-1/CCL2, significantly promoting macrophage infiltration and adipose tissue fibrosis (Halberg et al., 2009; Sun et al., 2013). Together, these findings demonstrate the essential role of the vasculature in adipose tissue expansion. Healthy adipose tissue growth induces angiogenesis to initiate hyperplastic expansion, whilst adipocyte hypertrophic growth is associated with insufficient angiogenesis, inducing hypoxia which in turn exacerbates unhealthy fat expansion.

### Future perspectives

Healthy adipocyte cell growth relies on the remodeling of multiple cellular functions to achieve cell growth and homeostasis. An unsuccessful adaptive regulation of cell growth is closely associated with hypertrophic adipocyte dysfunction. A recent study demonstrated that blunted catecholamine stimulated lipolysis persists even 5 years following Roux-en-Y gastric bypass surgery (RYGB), despite a significant reduction in fat cell size and marked weight reduction to nonobese levels (Ryden et al., 2022). Similar results were found by Spalding and colleagues who showed that lipid removal rate remained unchanged in obese individuals 2 years following gastric bypass surgery, despite significant weight loss (Arner et al., 2019). Such persistent dysregulated lipolysis may help explain poor long-term outcomes following weight loss. Patients with an increased lipolytic response to catecholamine stimulation pre-surgery, have a better likelihood of maintaining weight loss following bariatric surgery (Ryden et al., 2022). This data suggests lipolysis has a profound effect on regulating metabolic health and identifies lipolysis as a potential target for weight management. Nevertheless, stimulating adipocyte lipolysis may result in unnecessary elevated levels of serum fatty acids. Released fatty acids, if not oxidized for energy expenditure, may be taken back up and re-esterified for triglyceride synthesis. As such, therapeutic interventions that stimulate lipolysis could benefit from coupling this to the consumption of fatty acids through oxidation or energy wasting processes.

Recent studies identified ADRB3 as a potential target for adipocyte lipolysis (Finlin et al., 2020; O'Mara et al., 2020; Cero et al., 2021; Byun et al., 2022; Ryden et al., 2022; Valentine et al., 2022). ADRB3 activation has been shown to stimulate adipocyte lipolysis and induce a thermogenic program in both mouse and human adipocytes (O'Mara et al., 2020; Cero et al., 2021; Byun et al., 2022). In recent clinical trials, individuals with obesity and insulin resistance demonstrated improved oxidative metabolism and ameliorated glucose homeostasis following mirabegron (an ADRB3 agonist) treatment (Finlin et al., 2020; O'Mara et al., 2020; Cero et al., 2021; Byun et al., 2022; Valentine et al., 2022). Whilst encouraging, the effects of ADRB3 agonists tested so far are considered modest and short lived, partly due to reduced ADRB3 expression and catecholamine resistance in the adipose tissue of individuals with obesity (Valentine et al., 2022). These findings, however, demonstrate the potential for targeting adipocyte lipolysis as a mechanism to combat obesity-related disorders.

Understanding the altered membrane dynamics associated with enlarged adipocytes, including ion gated membrane channels and caveolae, may provide important insights into how to regulate the healthy expansion of fat cells and prevent hypertrophic related pathology. For example, upregulation of adipocyte SWELL1 protein expression using a small molecule approach (SN-401) activates SWELL1-dependent insulin signaling in adipocytes and improves systemic insulin sensitivity and tissue glucose uptake in mice with obesity and diabetes (Gunasekar et al., 2022). Given the sensitivity of caveolae dynamics to membrane lipid composition, a focus on dietary lipid composition could have important impacts on caveolae-mediated lipid uptake and healthy adipocyte cell expansion.

An increasing number of studies demonstrate the role of mTOR signalling in regulating cell growth and metabolic homeostasis (Gwinn et al., 2008; Arif et al., 2017; Ejaz et al., 2017; Xiong et al., 2018; Rivera-Gonzalez et al., 2020; Andrade et al., 2021; Fumagalli and Pende, 2022). Adipose stromal cells have been shown to hyper-activate Akt-mTOR signalling through GTP-binding RAS-like 3 (DIRAS3) knockdown, driving premature senescence in adipocyte progenitor cells and in differentiated adipocytes (Ejaz et al., 2017). Metformin, a well-known 5′ AMP-activated protein kinase (AMPK) activator effectively protects against human adipocyte senescence *in vitro*, by suppressing the AKT-mTOR pathway and inhibiting insulin-induced cell cycle entry (Li et al., 2021). Targeting the AMPK-mTOR pathway may thus be of therapeutic interest in alleviating hypertrophic-associated adipocyte dysfunction (Bailey et al., 2022; Ma et al., 2022). A newly discovered small molecule, aldometanib, mimics the role of glucose starvation and actives lysosomal-specific AMPK (Zhang et al., 2022). Aldometanib has been shown to inhibit mTORC1 in multiple tissues including muscle, liver and adipose tissue (Zhang et al., 2022). One month treatment of aldometanib in obese mice reduces blood glucose levels, alleviates fatty liver and reduces total fat mass (Zhang et al., 2022). The successful translation of aldometanib from rodent studies to the clinic remains to be seen.

Cell cycle regulation is a new and potentially important mechanism regulating adipocyte cellular growth. Animal studies with adipocyte-specific CDK4 or CDK5 knock-out/down exhibit reduced fat mass (Lagarrigue et al., 2016) and impaired insulin sensitivity (Banks et al., 2015). As previously discussed cell cycle entry in human mature adipocytes associates with increased cell and nuclear size (Li et al., 2021), yet cell cycle entry in fully differentiated 3T3L1 cells associates with reduced cell size, enhanced lipolysis and improved insulin sensitivity (Bailey et al., 2022). Clearly further studies are needed in this interesting area of emerging adipocyte biology. Jiang and colleagues show a link between cell cycle entry and beige (brown-like white adipocyte) adipocyte proliferation. The authors report a population of UCP1-positive adipocytes in white adipose tissue can turn on a proliferative program to generate new beige adipocytes, when stimulated by β3-adrenergic agonist (Park et al., 2021). Together, these findings demonstrate that cell cycle entry can occur in post-mitotic mature adipocytes, impacting multiple cellular functions. Additional studies are needed to fully elucidate the role of cell cycle entry, in facilitating/hindering adipocyte function. In summary, understanding the mechanisms of healthy adipocyte cell expansion may shed important insights into the processes underlying pathological adipose tissue expansion and define novel targets for therapeutic intervention in obesity-related disorders.
