# Supplementary material for: The regulation of adipocyte growth in white adipose tissue
Source: Front Cell Dev Biol. 2022 Nov 22;10:1003219. doi: 10.3389/fcell.2022.1003219 (PMC9723158; doi:10.3389/fcell.2022.1003219)
Supplement: Supplementary file 1 [file DataSheet1.PDF]

Box 1

White adipose tissue fat cells contain a large unilocular lipid droplet, predominately comprised of triglyceride, which occupies more that 90% of the fat cell volume. As such, changes in the amount of triglyceride stored and released from fat cells has significant impact on the size (and function) of the fat mass. The processes of synthesizing triglyceride in the fat cell (lipogenesis) as well as breaking down fat cell lipids (lipolysis) are briefly described in this box.

Adipocyte lipogenesis

Circulating triglycerides synthesized in the liver and intestine are packaged in the form of lipoproteins and travel to white adipose tissue. In adipose tissue, triglycerides are first hydrolyzed to non-esterified fatty acids (NEFA) at the surface of capillary endothelial cells via lipoprotein lipase (LPL). Next, NEFAs move through the endothelial lumen and are taken up by adipocytes through fatty acid transporters such as the scavenger receptor CD36 and caveolins. Aside from exogenous fatty acids, adipocytes also make use of glucose transported into fat cells to synthesize endogenous fatty acids through *de novo* lipogenesis (DNL). Exogenously and endogenously derived fatty acids are catalyzed to acyl-CoA and esterified to glycerol-3-phosphate (G3P) to form triglycerides.

G3P can be formed by the reduction of dihydroxyacetone phosphate or from glycerol recycling. Dihydroxyacetone phosphate derives from glucose or non-carbohydrate sources. Glucose transported intracellularly can be phosphorylated and converted to dihydroxyacetone phosphate via the glycolytic pathway. When cellular glucose levels are low, as in starvation or following a low-carbohydrate diet, dihydroxyacetone phosphate is converted mainly from non-carbohydrate precursors, including pyruvate, lactate and alanine, to generate G3P. This process is called glyceroneogenesis. The crucial regulatory step in glyceroneogenesis is mediated by phosphoenolpyruvate carboxykinase 1 (PCK1) which catalyzes oxaloacetate to phosphoenolpyruvate, which

is then converted to G3P. Glycerol recycling occurs when liberated glycerol, originally released during lipolysis, is phosphorylated by glycerol kinase to form G3P and recycled for triglyceride synthesis. The membrane glycerol channel aquaporin 7 (AQP7) facilitates glycerol efflux and regulates intracellular glycerol levels (Maeda et al., 2004).

Adipocyte lipolysis

During lipolysis, triglycerides in the lipid droplet are hydrolyzed to glycerol and three molecules of fatty acids. This is controlled via the action of three lipases: adipose triglyceride lipase (ATGL), hormone-sensitive lipase (HSL) and monoacylglycerol lipase (MGL). FAs released into the circulation can be taken up by skeletal muscle, brown adipocytes, liver and kidney for mitochondrial β-oxidation to fulfill energy demands. Released FAs can also travel back to the liver to be used as a major source of substrate for producing very-low-density lipoproteins (VLDLs).

Maeda, N., Funahashi, T., Hibuse, T., Nagasawa, A., Kishida, K., Kuriyama, H., et al. (2004). Adaptation to fasting by glycerol transport through aquaporin 7 in adipose tissue. *Proc Natl Acad Sci U S A* 101(51), 17801-17806. doi: 10.1073/pnas.0406230101.
